# Sentiment analysis of COP9-related tweets: a comparative study of pre-trained models and traditional techniques

**DOI:** 10.3389/fdata.2024.1357926

**Published:** 2024-03-20

**Authors:** Sherif Elmitwalli, John Mehegan

**Affiliations:** Tobacco Control Research Group, Department for Health, University of Bath, Bath, United Kingdom

**Keywords:** sentiment analysis, lexicon-based, Bi-LSTM, BERT, GPT-3, COP9, LLMS

## Abstract

**Introduction:**

Sentiment analysis has become a crucial area of research in natural language processing in recent years. The study aims to compare the performance of various sentiment analysis techniques, including lexicon-based, machine learning, Bi-LSTM, BERT, and GPT-3 approaches, using two commonly used datasets, IMDB reviews and Sentiment140. The objective is to identify the best-performing technique for an exemplar dataset, tweets associated with the WHO Framework Convention on Tobacco Control Ninth Conference of the Parties in 2021 (COP9).

**Methods:**

A two-stage evaluation was conducted. In the first stage, various techniques were compared on standard sentiment analysis datasets using standard evaluation metrics such as accuracy, F1-score, and precision. In the second stage, the best-performing techniques from the first stage were applied to partially annotated COP9 conference-related tweets.

**Results:**

In the first stage, BERT achieved the highest F1-scores (0.9380 for IMDB and 0.8114 for Sentiment 140), followed by GPT-3 (0.9119 and 0.7913) and Bi-LSTM (0.8971 and 0.7778). In the second stage, GPT-3 performed the best for sentiment analysis on partially annotated COP9 conference-related tweets, with an F1-score of 0.8812.

**Discussion:**

The study demonstrates the effectiveness of pre-trained models like BERT and GPT-3 for sentiment analysis tasks, outperforming traditional techniques on standard datasets. Moreover, the better performance of GPT-3 on the partially annotated COP9 tweets highlights its ability to generalize well to domain-specific data with limited annotations. This provides researchers and practitioners with a viable option of using pre-trained models for sentiment analysis in scenarios with limited or no annotated data across different domains.

## 1 Introduction

Social media platforms have gained significant prominence as sources of information, opinions, and emotional expressions. Among these platforms, Twitter has witnessed a surge in popularity, serving as a space for individuals to voice their thoughts on various subjects, encompassing entertainment, politics, and social issues. The analysis of Twitter sentiments holds the potential to unveil collective perspectives, preferences, and attitudes within groups (Singh et al., 2020). Furthermore, the comparison of sentiment patterns among diverse demographic segments, including people from different countries, age groups, backgrounds, or genders, offers valuable insights into how individuals from varied contexts perceive and respond to events, products, or policies (Gulati et al., 2022).

Sentiment analysis plays a pivotal role for businesses and organizations aiming to gauge the public perception of their products, services, or campaigns. By ascertaining the sentiment conveyed in tweets related to their brand or industry, they can pinpoint areas for enhancement, monitor public sentiment, and adapt their marketing strategies accordingly. Additionally, sentiment analysis provides a deeper understanding of human behavior, public opinion, and marketing tactics, aiding in the recognition of trends, patterns, and variations in attitudes across different segments of the population. This, in turn, leads to more effective communication, marketing, and policy formulation (Lin et al., 2020).

Given the imperative to prioritize tobacco control objectives and devise clear strategies for their implementation has led to the selection of this research's focus (Myers Smith et al., 2021). The World Health Organization's Framework Convention on Tobacco Control (WHO FCTC) emerged in response to the global spread of the tobacco epidemic. It convenes a biennial Conference of the Parties (COP) to review and advance the implementation of the convention and adopt amendments, protocols, or annexes as necessary (Bialous and e Silva, 2022). Analyzing the Twitter discourse surrounding COP meetings offers valuable insights into the public's perception of COP and endeavors to shape the narrative surrounding its activities.

Despite extensive research in sentiment analysis, a systematic comparison of traditional machine learning approaches with advanced deep learning models like Bidirectional Encoder Representations from Transformers (BERT) and Generative Pre-trained Transformer 3 (GPT-3) specifically for Twitter sentiment analysis remains limited. This study aims to fill this gap by conducting a comparative evaluation of various techniques applied to tweets, seeking to identify the most suitable approach within this domain. Analyzing sentiments expressed in tweets related to events like the COP9 conference can provide valuable insights for stakeholders, hence the importance of identifying high-performing sentiment analysis methods for tweets. However, the informal language and short length of tweets poses challenges compared to other domains, necessitating a targeted analysis. By applying both standard tweet datasets and real-world COP9 data, this study seeks to examine the nuances of diverse techniques for accurate Twitter sentiment analysis especially in scenarios with limited or no annotated data.

Therefore, this research endeavors to fulfill several objectives. It seeks to assess and compare the performance of various sentiment analysis techniques using datasets from IMDB reviews and Sentiment140. Through the evaluation of these techniques using standard metrics like accuracy, F1-score, and precision, it aims to pinpoint the most effective approach. Subsequently, the study applies the top-performing sentiment analysis model to scrutinize the sentiments expressed in tweets related to COP9. The findings may guide future research and applications of sentiment analysis on social media from diverse domains.

## 2 Related work

Sentiment Analysis proved to be very effective in detecting the underlying tone of the analyzed content. Many of the published studies have focused on sentiment analysis of Twitter messages, mainly because a large and diverse population expresses opinions about almost any topic daily on this platform. Several approaches are used in sentiment analysis in different domains. A study by Jain et al. (2021) conducted a systematic literature review to compare, analyze, explore, and identify research gaps related to consumer sentiment analysis in the domain of hospitality and tourism. The study examined the use of statistical analysis techniques in analyzing online reviews and employed word frequency-based approaches to identify several types of sentiment.

According to a study conducted by Al-Natour and Turetken (2020) that applied rule-based techniques, contextual factors, such as product type and review length, were found to influence the ability of a technique to accurately reflect the genuine sentiment of a review. Multiple lexicon-based sentiment analysis techniques were examined in the research, with Vader achieving marginally superior performance.

In another investigation by Zhang et al. (2019), the authors examined the use of deep learning for solving sentiment analysis tasks, specifically in relation to sentiment polarity. The study utilized datasets such as Sentiment140 and IMDB Reviews, employing both Term Frequency-Inverse Document Frequency (TF-IDF) and word embedding techniques. The comparative analysis yielded maximum accuracies of 0.82 and 0.87 when using Recurrent Neural Network (RNN) and word embedding approaches, respectively, on Sentiment140 and IMDB Reviews datasets.

Moreover, a study by Wankhade et al. (2022) presented a comprehensive review of sentiment analysis methods, evaluating and comparing several approaches, including Lexicon-based, Machine Learning, Hybrid, and Transfer Learning. The study achieved a maximum accuracy of 0.90 for IMBD reviews and 0.82 for Sentiment140 using an Attention-based Bidirectional CNN-RNN Deep Model.

In a research paper published by Zhao et al. (2021), an approach was proposed a deep learning technique that combined Convolutional Neural Network (CNN) with Gated Recurrent Unit (GRU), leveraging the local features generated by CNN and the long-term dependency learned by GRU. The proposed technique was evaluated on datasets comprising reviews of hotels and cars, demonstrating comparable accuracy performance of Long Short-Term Memory (LSTM), CNN, and Support Vector Machines (SVM) through a series of experiments.

A recent study conducted by Wadawadagi and Pagi (2020) examined the effectiveness of various deep learning architectures for sentiment classification. Four distinct publicly available datasets were utilized to measure the accuracy of both binary and fine-grained sentiment classification models. Global Vectors for Word Representation (GloVe) was employed as a word embedding technique, and a range of deep learning approaches, including CNN, RNN, and several advanced techniques, were tested. The results indicated that the CNN model achieved the highest accuracy of 0.82 in product reviews, while the RNN model attained 0.69 accuracy in analyzing tweets. Furthermore, research by Wang et al. (2020) employed trend analysis and thematic analysis to identify negative sentiment characteristics. The fine-tuned BERT achieved sentiment classification with considerable accuracy of 0.76 regarding COVID-19. The results derived from social media posts provided valuable guidance on public health responses that could alleviate public concerns.

Various sentiment analysis techniques, including lexicon-based, Machine Learning (ML), and deep learning approaches, have been evaluated in a recent study (Rodríguez-Ibánez et al., 2023). Among these, BERT demonstrated superior performance, particularly in sentiment analysis of IMDB reviews and social networks. The study delved into diverse domains where these techniques have found application, including marketing, politics, economics, and health. However, it noted limited usage in specific areas, such as emergencies. Concurrently, another study by Tiwari et al. (2023) highlighted machine learning and ensemble learning namely bagging-based and boosting-based ensemble techniques as popular and effective mechanisms for sentiment analysis, offering valuable insights into prevalent practices and major publishing platforms. However, the study lacked a detailed comparison of various sentiment analysis techniques.

Building on these findings, recent studies have explored leveraging BERT frameworks for sentiment analysis on tweets. A study by Wan et al. (2024) proposed enhancing BERT with emotion-cognitive reasoning to improve feature representation and sentiment classification performance on emergency-related tweets. However, the approach relied on simplified emotion rules and emotion word lexicons. Research by Pota et al. (2021) focused on evaluating different pre-processing strategies applied to informal Twitter text before using BERT for sentiment analysis in English and Italian. The results provided insights into optimal tweet pre-processing to boost BERT's performance. Another study by Bello et al. (2023) combined BERT's contextual embeddings with deep learning classifiers like CNN and Bidirectional LSTM (Bi-LSTM), training the model on multiple tweet datasets demonstrating BERT's capabilities. The research suggested investigating other transformer models as well. These relevant works showcase BERT's effectiveness for sentiment analysis of informal, contextual tweets when combined with strategies like pre-processing, emotion-cognitive rules, and deep learning classifiers. The studies recommend future enhancements such as newer language models, multilingual approaches, and exploring different transformer architectures.

Another research by Zhang et al. (2020), highlighted the challenge posed by the lack of annotated datasets that can hinder the effective training of deep learning models. The study proposed three transfer learning techniques for analyzing public sentiment toward HPV vaccines on Twitter, which incorporated Embeddings from Language Model (ELMo), BERT, and GPT models. The fine-tuned BERT model, however, demonstrated superior performance when compared to the other approaches. According to a study conducted by Zhao and Yu (2021), incorporating external domain-specific knowledge can be a beneficial strategy for overcoming the limitations associated with insufficient training data. The researchers proposed injecting sentiment domain knowledge into the language representation model and demonstrated the effectiveness of this approach. The results indicated that the knowledge-enabled BERT model is a promising solution for addressing aspect-based sentiment analysis problems.

A study conducted by Farha and Magdy (2021) focused on Arabic sentiment analysis, exploring methodologies, experimental setups, and the necessity for advancements in Arabic sarcasm detection. This study advocated for deep learning, placing particular emphasis on the importance of sarcasm detection in Arabic Natural Language Processing (NLP). The study centered on LSTM and BERT while also suggesting the promising performance of pre-trained Language Models. However, they emphasized that annotated Arabic SA datasets have limitations and challenges. Furthermore, in a separate study (Omar et al., 2021), the authors developed a standard multi-label Arabic dataset through a combination of manual annotation and a semi-supervised annotation technique. In another investigation, Van Thin et al. (2023) critically examined pre-trained language models for sentiment analysis in Vietnamese, endorsing PhoBERT. This study addressed challenges, such as input length limitations, by proposing truncation methods and recommending the utilization of generative models.

Therefore, the existing research in sentiment analysis faces a common limitation of dealing with insufficient annotated datasets for training deep learning models. This scarcity of labeled data hinders the effective training and performance of these models, potentially leading to suboptimal results and reduced accuracy in sentiment analysis tasks.

The primary contribution of this research is a thorough examination of sentiment analysis approaches across several datasets, with a key aim being to address the persistent challenge of insufficiently annotated datasets for the effective training of deep learning models. The research systematically compares common methodologies, specifically categorized into Lexicon-based, Machine Learning, and Deep Learning, with a particular emphasis on advanced Transformer models like BERT and GPT-3. The evaluation encompasses two commonly used datasets for Tweet sentiment analysis, namely IMDB reviews and Sentiment140, shedding light on the performance nuances of each technique. Additionally, the exploration of the impact of text or tweet features on model performance is undertaken. Notably, real-world challenges are addressed, exemplified by the WHO Framework Convention on Tobacco Control Ninth Conference of the Parties in 2021 (COP9) tweets. The application of the best-performing sentiment analysis model, initially validated on partially annotated COP9 data, to the entire COP9 Tweets dataset is undertaken, providing valuable insights for practitioners and researchers actively seeking effective sentiment analysis techniques in diverse domains.

The remainder of this paper is organized as follows. Section 3 presents the framework of sentiment analysis approaches used in the field of NLP and sentiment analysis. Section 4 presents the results of the applied techniques on the standard datasets followed by the assessment of those techniques on partially annotated COP9 data. The final section presents conclusions about the differences between the utilized sentiment analysis techniques.

## 3 Methods

**Figure 1:**
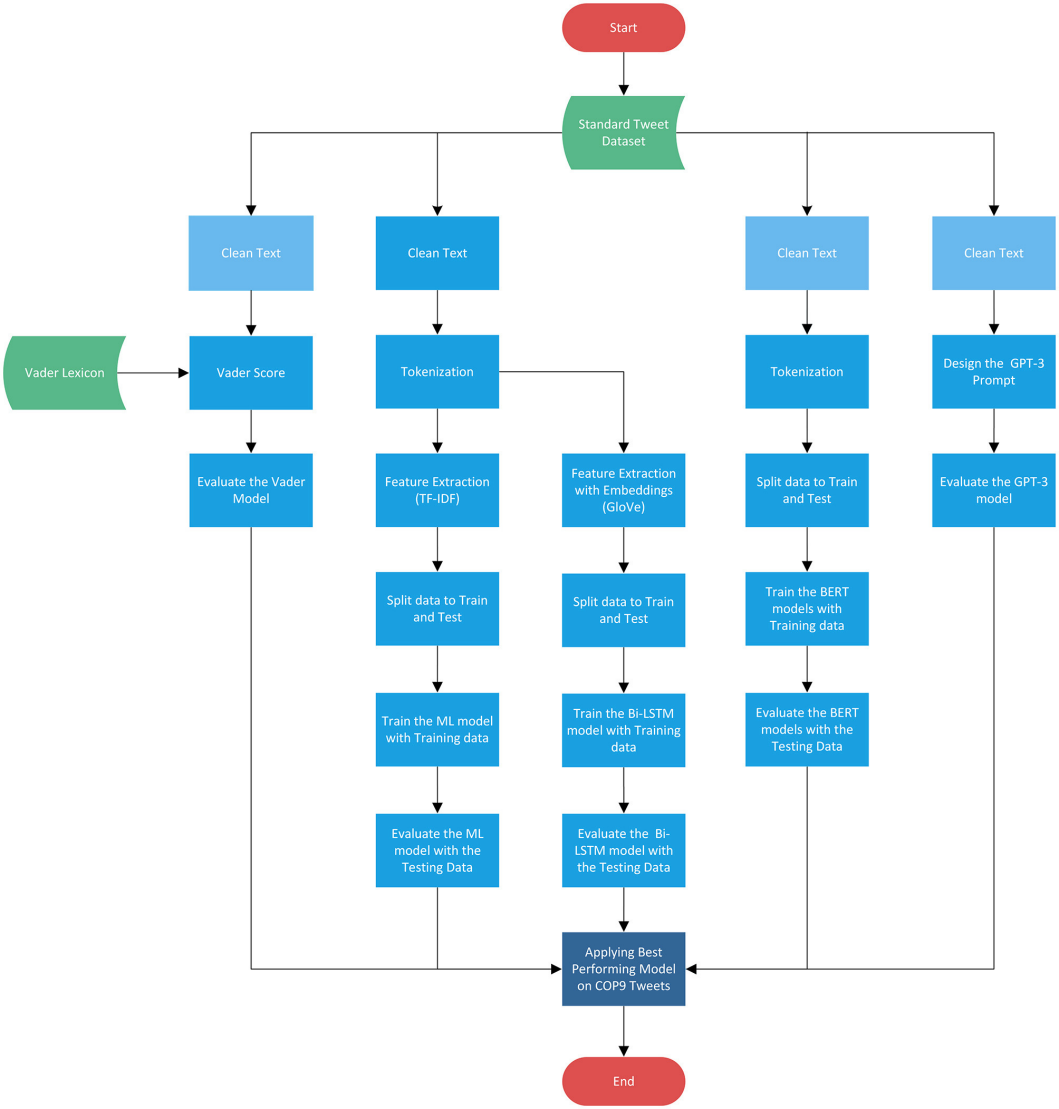
The methodology flowchart for the sentiment analysis of COP9 tweets.

The methodology employed in this research, as depicted in Figure 1, encompasses five distinct approaches for sentiment analysis: Lexicon-based, Machine Learning, Bi-LSTM, BERT, and GPT-3. Among these approaches, Vader, BERT, and GPT-3 exhibit relatively lower dependence on intensive text cleaning, unlike ML and Bi-LSTM, which may necessitate some level of text preprocessing to eliminate potential noise, particularly in the context of tweets. Therefore, it is recommended to apply appropriate text preprocessing techniques to enhance the quality of the data before subjecting it to the sentiment analysis pipeline.

The proposed methodology begins with the application of multiple sentiment analysis models to two widely used standard datasets for tweets sentiment analysis. Subsequently, the top-performing models are tested on manually annotated COP9 tweets to identify the most effective one. Finally, the sentiment analysis of COP9 tweets is conducted using the selected best performing model. This comprehensive approach aims to ascertain the optimal sentiment analysis model for COP9 tweets and gain valuable insights into the sentiments expressed during the conference.

The subsequent subsections will delve into the various processing units associated with the sentiment analysis pipeline, elucidating the specific methods and steps undertaken to effectively analyze and interpret sentiments from the collected data.

### 3.1 Datasets

The first dataset used in the study is the IMDB reviews dataset, which is a collection of movie reviews acquired from the Internet Movie Database (IMDB). The dataset is widely used in NLP research and machine learning tasks, particularly sentiment analysis. It has 50,000 movie reviews, each of which is labeled either “positive” or “negative” and is written in English. The reviews include a wide range of films, including drama, action, comedy, and horror (Topal and Ozsoyoglu, 2016).

The second dataset used is the Sentiment140 dataset which is a collection of tweets that have been labeled as either positive or negative for sentiment analysis. The dataset was created by Stanford University and is widely used in research for NLP and machine learning tasks. The dataset contains 1.6 million tweets that were obtained from Twitter between April 2009 and June 2009. The tweets in the Sentiment140 dataset are in English and cover a variety of topics, including politics, sports, entertainment, and more. One advantage of the Sentiment140 dataset is its large size, which allows for more robust model training and evaluation. Overall, the Sentiment140 dataset is a valuable resource for researchers and developers working on NLP and machine learning tasks related to sentiment analysis (Go et al., 2009).

The IMDB dataset provides longer-form movie reviews with clearly expressed sentiments, while Sentiment140 consists of short informal tweets. Using both datasets enables comparing model performance on varying text lengths—the concise nature of tweets poses greater challenges compared to longer movie reviews. Furthermore, both datasets are widely benchmarked for sentiment analysis research, enabling standardized comparisons to previous studies (Tan et al., 2023). The labels are limited to positive and negative, aligning with the binary sentiment analysis employed in the first stage of this work. Although movie and tweet domains differ from COP9, these datasets establish baseline model capabilities on standard sentiment analysis tasks before testing on COP9 tweets. The IMDB and Sentiment140 datasets provide the means to thoroughly assess different techniques on diverse text types relevant to analyzing sentiments in tweets.

The third dataset comprises COP9 tweets collected using the Digital Methods Initiative Twitter Capture and Analysis Toolkit (DMI-TCAT), focusing on tweets relevant to COP9. DMI-TCAT enables continuous tweet collection through Twitter's Streaming API, offering a representative sample in proportion to the total volume of tweets posted at any given time (Groshek et al., 2020). Tweets were gathered containing the hashtags #COP9 and #COP9FCTC between 07/11/2021 and 22/11/2021, resulting in a dataset of 7,377 tweets (Elmitwalli et al., 2024). To guarantee a cohesive English analysis of COP9 tweets, the Google Translate API was employed, ensuring accurate understanding of the content (Banik et al., 2019). The dataset duplicate tweets were filtered out before any processing. While 30% of the tweets were manually annotated for the assessment stage. Moreover, to ensure the accuracy of sentiment labels, we collaborated with three experts specializing in Tobacco Tactics. Their expertise in tobacco-related discourse enabled precise categorization of tweets based on sentiment.

### 3.2 Text cleaning

Text cleaning is a crucial step in sentiment analysis, aimed at improving the accuracy and reliability of results. Common cleaning steps include removing stop words, converting text to lowercase for consistency, eliminating punctuations, numbers, and special characters, as well as applying stemming and lemmatization to reduce word forms. Additionally, it involves removing URLs, user mentions, and correcting misspelled words. In the context of social media data, emoji replacement can be used to enhance sentiment understanding. These actions collectively reduce noise and irrelevant information, ultimately ensuring the quality of sentiment analysis outcomes (Kit and Mokji, 2022).

### 3.3 Tokenization

Tokenization is a crucial step in sentiment analysis, as it breaks down text into smaller units called tokens, typically words or sub-words. This process transforms unstructured text data into a structured format suitable for analysis by machine learning algorithms (Gaye et al., 2021). There are several key considerations to keep in mind when tokenizing text data for sentiment analysis. Word-level tokenization is the most widely used approach in sentiment analysis, where the text is segmented into individual words. This allows the model to assess the sentiment of individual words and phrases, contributing to the determination of the overall sentiment of the text. In some cases, subword-level tokenization can be employed to enhance the accuracy of sentiment analysis. This technique involves breaking words into smaller subunits, which can be particularly beneficial in languages with complex inflections or when dealing with out-of-vocabulary words. Furthermore, specific types of text data may require custom tokenization techniques. For instance, social media content may need unique handling of emoticons, hashtags, and mentions.

### 3.4 Feature extraction

Feature extraction in NLP plays a vital role by converting text into numerical features suitable for machine learning and deep learning techniques (Lastra-Díaz et al., 2019). Two common approaches to feature extraction are word frequency-based and word embedding-based methods, each offering distinct advantages in sentiment analysis. Word frequency approaches, which include techniques like Bag of Words (BoW), Term Frequency-Inverse Document Frequency (TF-IDF), and N-grams, focus on representing text using word counts and word associations without considering context. BoW creates a vocabulary of unique words and represents each document as a vector of word counts, while TF-IDF calculates the importance of words in the context of the entire corpus (Onan, 2021). N-grams are used to capture word sequences and enhance sentiment analysis by identifying patterns and context in text data. On the other hand, word embedding approaches, such as Word2Vec, FastText, and GloVe, encode words as dense vectors in high-dimensional spaces. These techniques capture the semantic and syntactic meaning of words (Pimpalkar, 2022; Aoumeur et al., 2023; Umer et al., 2023), making them effective in sentiment analysis, especially for handling idiomatic expressions, sarcasm, and figurative language. Word2Vec, for instance, trains a neural network on a large text corpus to generate word vectors, enabling accurate word similarity calculations and the improvement of sentiment analysis models. FastText, which relies on character n-grams, captures sub-word information and effectively handles uncommon or unknown words. GloVe, using a co-occurrence matrix and singular value decomposition (SVD), captures global patterns of word co-occurrence and provides context-rich word embeddings.

Moreover, recent deep learning models like BERT and GPT use self-attention mechanisms to capture word context and generate contextualized embeddings. Unlike traditional word embeddings, these models directly incorporate contextual information into their architecture, making them well-suited for various sentiment analysis tasks without the need for explicit word embedding stages. Therefore, the choice between these feature extraction approaches depends on specific data characteristics and the needs of sentiment analysis tasks, offering flexibility and adaptability in handling text data for improved sentiment analysis results.

### 3.5 Sentiment analysis models

There are several approaches to design the sentiment analysis model, including:

#### 3.5.1 Lexicon-based approach

The lexicon-based approach for sentiment analysis relies on a predetermined list of words with associated sentiment scores to gauge the sentiment of a text. This method involves summing up the scores of individual words within the text to arrive at an overall sentiment assessment, making it a fast and efficient process, though it may lack the precision of other techniques as been illustrated in Algorithm 1.

**Algorithm 1:**
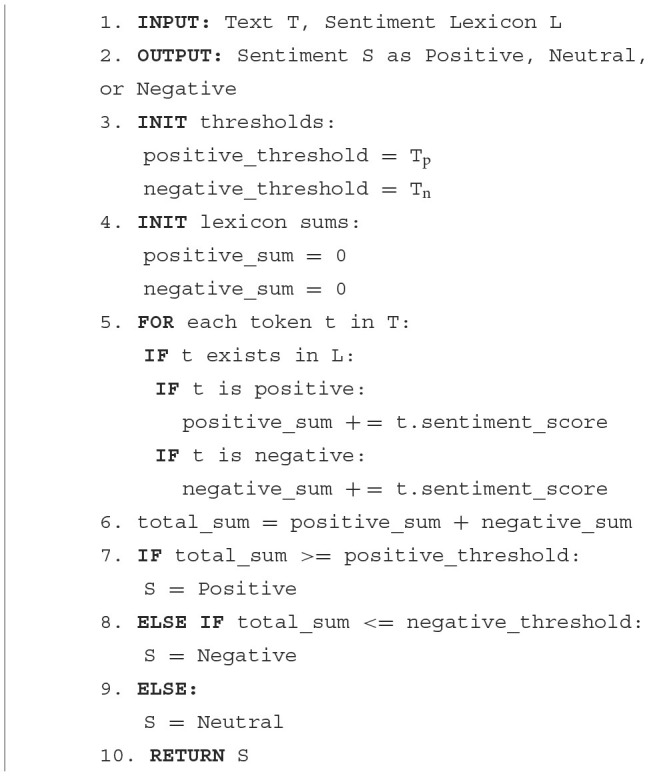
Lexicon-based sentiment analysis.

In this study, a comparative analysis was conducted employing VADER (Valence Aware Dictionary and sEntiment Reasoner), a lexicon-based sentiment analysis tool developed specifically for social media content, such as tweets (Wook et al., 2019). VADER is a rule-based system that evaluates sentiment using a predefined lexicon of words and phrases, each with assigned polarity scores. The VADER sentiment analysis process unfolds through several stages, including tokenization, where the tweet is divided into individual words and punctuation. Polarity scoring comes next, with each word assigned a polarity score, indicating its positive, negative, or neutral sentiment. Notably, VADER's sentiment lexicon contains over 7,500 words and phrases with polarity scores.

VADER's strength lies in its contextual analysis, considering the context in which words are used. It calculates a valence score by aggregating individual word scores, resulting in an overall sentiment score ranging from −1 (negative) to +1 (positive). VADER's effectiveness is further enhanced by its consideration of additional features like emoticons and punctuation, which can intensify the expressed sentiment, depending on their usage. In the end, VADER returns the sentiment score for the tweet, along with information regarding sentiment strength and classification as positive, negative, or neutral. Furthermore, VADER proves to be a quick and efficient approach for analyzing sentiment in Twitter data, making it a valuable tool for rapidly assessing the overall sentiment of a large volume of tweets related to a specific topic or event (Wook et al., 2019). In reference to a study in Hutto and Gilbert (2014), the authors investigated the classification accuracy, employing thresholds set at −0.05 and +0.05 for all normalized sentiment scores ranging between −1 and 1. The results showed that VADER (F1 = 0.96) outperformed individual human raters (F1 = 0.84) in accurately classifying tweet sentiments across four different domains.

#### 3.5.2 Machine learning approach

Machine learning models such as Logistic Regression, Naive Bayes, and Support Vector Machines rely on statistical approaches and algorithms that learn to make predictions from data. Therefore, this approach involves training a machine learning algorithm on a large dataset of labeled text to predict the sentiment of new, unlabelled text. It can be more accurate than the Lexicon-based approach but requires a large amount of labeled data to train the algorithm. There are several machine learning techniques commonly used in sentiment analysis including:

- Logistic regression: A statistical model for classification tasks in machine learning. Based on a collection of input variables, it predicts the likelihood of a binary result. The logistic function, which transforms any real-valued input to a value between 0 and 1, is used to change the output variable in logistic regression (Jaya Hidayat et al., 2022).
- Naive Bayes Classifier: A probabilistic algorithm that calculates the probability of a document belonging to a certain class (e.g., positive or negative) based on the frequency of words in the document. It is one of the simplest and most popular algorithms used for sentiment analysis (Gautam et al., 2021).
- Support Vector Machines (SVMs): A supervised machine learning algorithm used to classify data by finding a hyperplane that separates two classes of data with the maximum margin. SVMs are commonly used in sentiment analysis due to their ability to handle high-dimensional data and non-linear relationships between features (Kurani et al., 2021).
- Decision Trees: A supervised machine learning algorithm that creates a tree-like model of decisions and their possible consequences. Decision trees are commonly used in sentiment analysis to classify text based on a set of rules or conditions (Fitri et al., 2019).
- Random Forest: An ensemble learning method that combines multiple decision trees to improve the accuracy of predictions. Random forests have been shown to be effective in sentiment analysis due to their ability to handle noisy and high-dimensional data (Angadi and Reddy, 2021).
- Extra Trees, also known as Extremely Randomized Trees, is a tree-based ensemble learning algorithm. It is similar to Random Forest, but instead of choosing the best feature to split at each node, Extra Trees selects a random subset of features and chooses the best split among them. This randomness reduces overfitting and increases the diversity of the trees, which can lead to better generalization and improved accuracy (Aljedaani et al., 2021).
- K-Nearest Neighbors (KNN) is a non-parametric algorithm used for classification and regression. KNN works by finding the K closest data points to a given input data point and classifying it based on the majority class among those K neighbors. In sentiment analysis, KNN can be used to classify a text as positive or negative based on the similarity of its features to those of the labeled examples in the training dataset. The similarity is typically measured using a distance metric such as Euclidean distance or cosine similarity (Paul et al., 2022).
- Gradient Boosting: Another ensemble learning method that combines multiple weak learners to create a strong learner. Gradient boosting has been shown to be effective in sentiment analysis due to its ability to handle imbalanced datasets and improve the accuracy of predictions (Hama Aziz and Dimililer, 2021).

Generally, each of these machine learning techniques has its own strengths and weaknesses when applied to sentiment analysis. Choosing the appropriate technique depends on the specific application and the characteristics of the data being analyzed.

#### 3.5.3 Deep learning approach

Deep learning models such as LSTM, BERT, and GPT-3 use multi-layer neural networks capable of learning hierarchical representations and complex relationships in data. Therefore, the approach involves using deep neural networks to automatically learn features and patterns in the text for sentiment analysis. It has been shown to be effective in handling complex and large-scale data, but it requires a significant amount of computing power and data to train the models. There are several architectures that could be used effectively in sentiment analysis including Bi-LSTMs that are commonly used to capture contextual information from text in both forward and backward directions (Lin et al., 2021). BERT is a transformer-based neural network architecture that is pre-trained on large amounts of data and has been shown to be highly effective in various NLP tasks including sentiment analysis (Mann et al., 2023). BERT can understand the context of a sentence and can produce highly accurate predictions. GPT-3 is another powerful language model that can generate human-like text and can perform a wide range of NLP tasks, including sentiment analysis. Its large pre-trained model and ability to generate coherent text make it a promising tool for sentiment analysis. These models are further explained as follows:

a) Bi-LSTM

Bidirectional Long Short-Term Memory (Bi-LSTM) is a neural network architecture, representing a modified form of the Long Short-Term Memory (LSTM) network. This variant is designed to process inputs bidirectionally, allowing it to consider contextual information from both preceding and succeeding elements in a sequence. Like the traditional LSTM network, the Bi-LSTM can be trained on a labeled dataset, where each example comprises a piece of text and its corresponding sentiment label (Jang et al., 2019).

**Figure 2:**
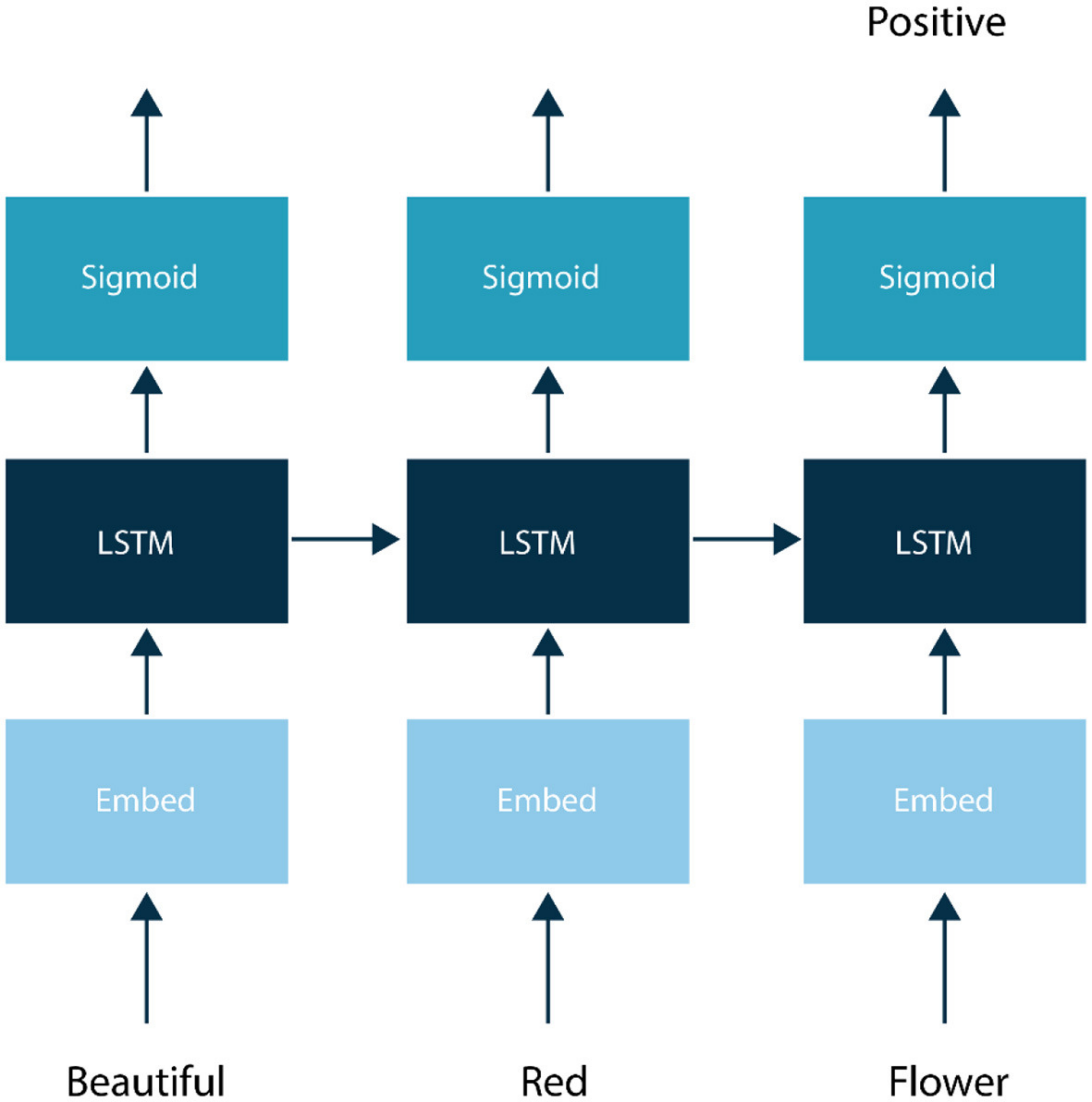
LSTM architecture for sentiment analysis.

The Bi-LSTM network consists of multiple layers, each of which contains multiple LSTM units. The input layer receives the text as a sequence of word embeddings (i.e., vector representations of the words) as shown in Figure 2. The LSTM layers then process the input sequence in both forward and backward directions, allowing the network to capture both the past and future context of each word. The outputs of the forward and backward LSTMs at each time step are concatenated and passed to a fully connected layer for classification. The output of the final layer represents the predicted sentiment label for the input text.

LSTM cells can be described using Equations (1–6) as follows:


(1) $$\begin{array}{lll} i_{t} & = & \sigma \left(x_{t}U^{i}+h_{t-1}W^{i}\right) \end{array}$$

(2) $$\begin{array}{lll} f_{t} & = & \sigma \left(x_{t}U^{f}+h_{t-1}W^{f}\right) \end{array}$$

(3) $$\begin{array}{lll} o_{t} & = & \sigma \left(x_{t}U^{o}+h_{t-1}W^{o}\right) \end{array}$$

(4) $$\begin{array}{lll} {\tilde{C}}_{t} & = & \tanh\left(x_{t}U^{g}+h_{t-1}W^{g}\right) \end{array}$$

(5) $$\begin{array}{lll} C_{t} & = & \sigma \left(f_{t}*C_{t-1}+i_{t}*{\tilde{C}}_{t}\right) \end{array}$$

(6) $$\begin{array}{lll} h_{t} & = & \tanh\left(C_{t}\right)*o_{t} \end{array}$$

Where *i*_*t*_ is the input gate. *f*_*t*_ is the forget gate, (${\tilde{C}}_{t}$ and *C*_*t*_) are cell status, while (*o*_*t*_ and *h*_*t*_) are the outputs.

One advantage of using a Bi-LSTM network for sentiment analysis is that it can capture the dependencies between words in the input sequence, which is important for understanding the context of each word in relation to the entire text. Additionally, the bidirectional processing allows the network to incorporate information from both the past and future, improving its ability to make accurate predictions (Omara et al., 2022).

b) BERT

**Figure 3:**
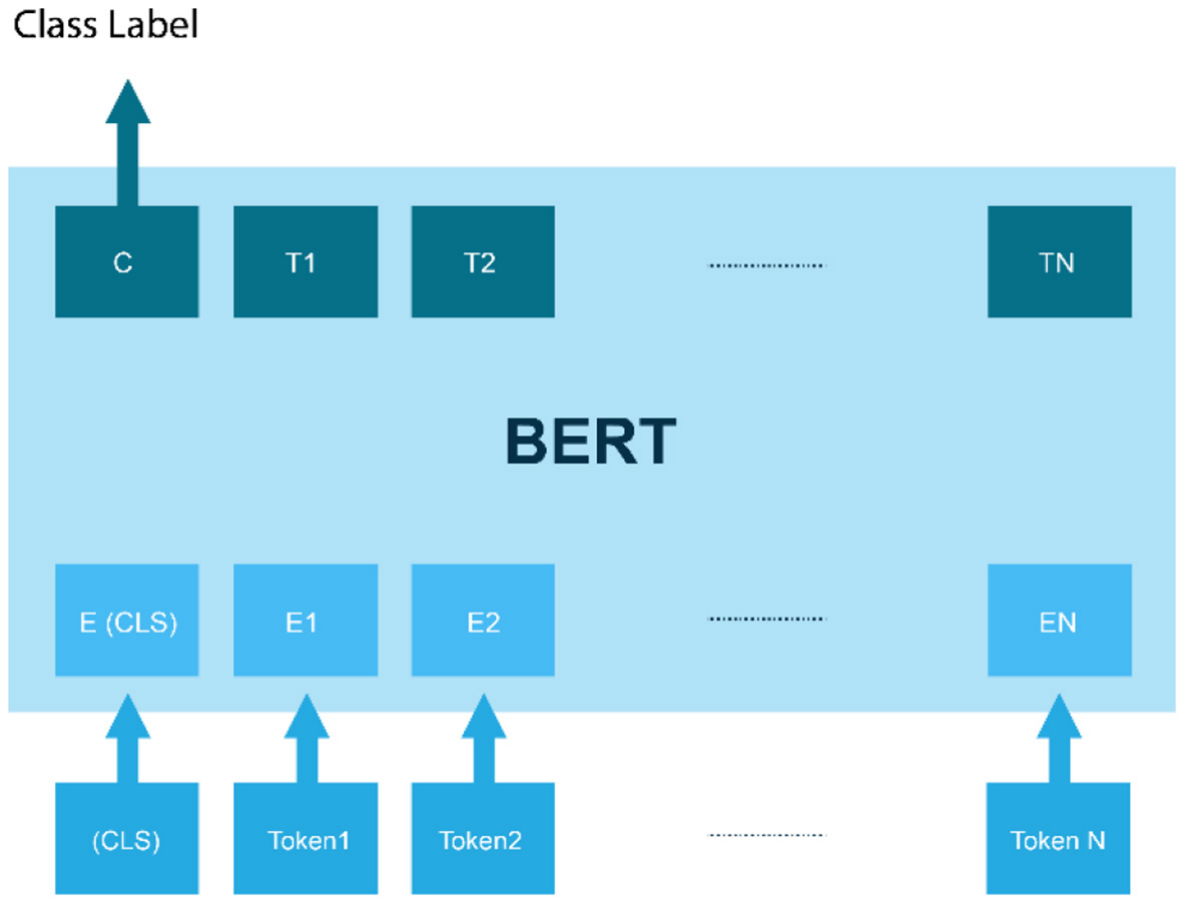
BERT architecture for sentiment analysis.

Bidirectional Encoder Representations from Transformers (BERT), a milestone deep learning model for NLP tasks, offers a powerful approach to sentiment analysis. BERT is a pre-trained language model that can be adapted for specific NLP tasks, including sentiment analysis, with only a relatively small amount of additional training data (Alaparthi and Mishra, 2021). BERT's architecture comprises multiple Transformer layers, specialized for processing sequential data like text. It tokenizes input text into subword units and processes it bidirectionally, considering both preceding and following context, enabling the model to capture long-term dependencies and context more effectively than previous models as shown in Figure 3. This model is initially trained using Masked Language Modeling (MLM), an unsupervised pre-training approach (Zhao and Yu, 2021). During this phase, BERT predicts masked words in sentences, with its encoder incorporating self-attention mechanisms to understand word relationships within sentences (Pota et al., 2021). For sentiment analysis, BERT is fine-tuned on labeled datasets, allowing it to learn both token-level semantics and sentiment labels. BERT's advantage lies in its ability to capture intricate word and sentence relationships, making it adept at understanding nuanced text meaning. Its pre-training on a vast text corpus also enables effective generalization to new tasks, reducing the need for extensive labeled data (Khan et al., 2022).

c) GPT-3

Generative Pre-trained Transformer 3 (GPT-3) is a language model developed by OpenAI, which has gained attention for its impressive ability to generate coherent and natural language text. GPT-3 is pre-trained on a large corpus of text data and can be fine-tuned on a wide range of NLP tasks, including sentiment analysis (Liu et al., 2023).

**Figure 4:**
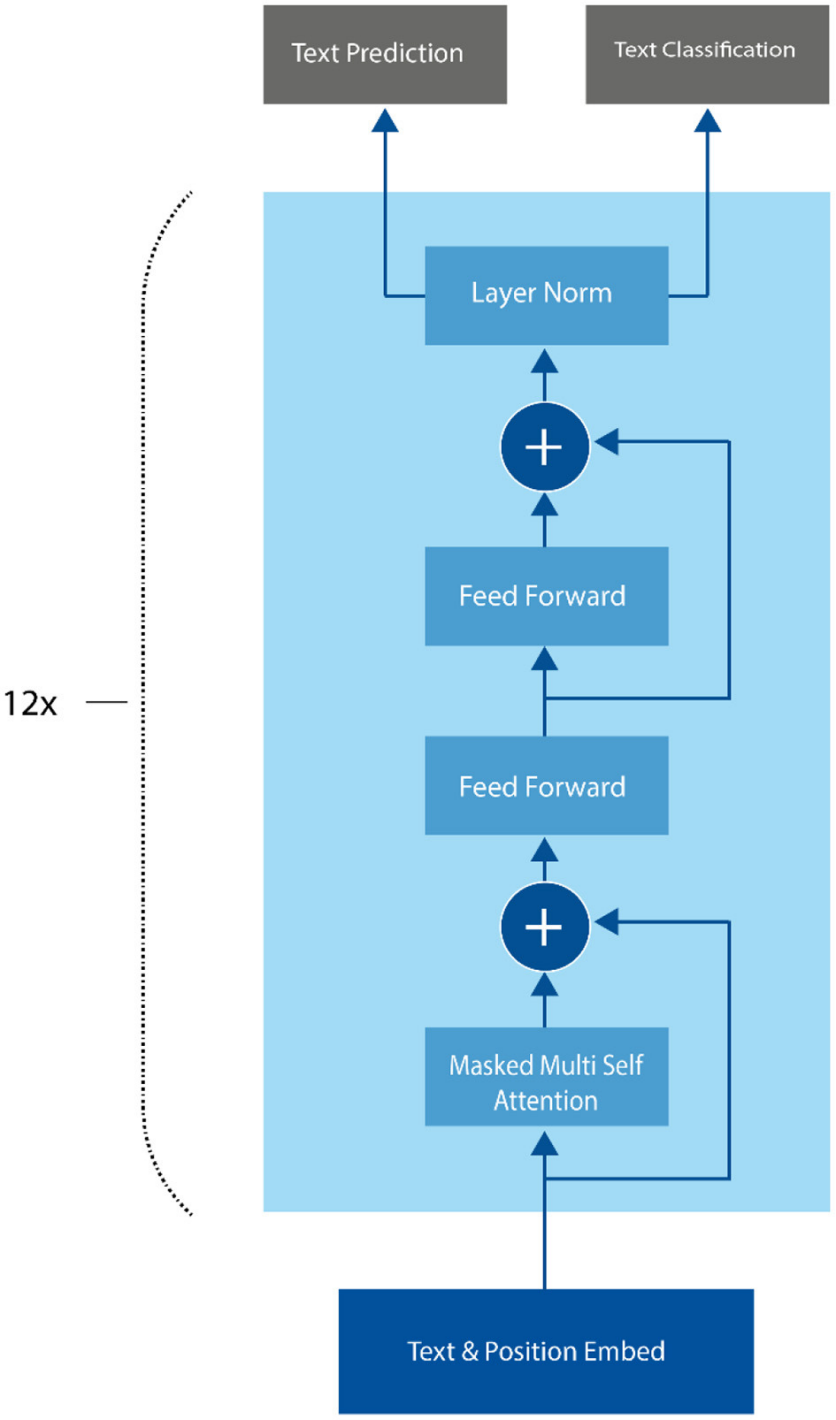
GPT-3 architecture.

In sentiment analysis, GPT-3 can be fine-tuned on a labeled dataset of text examples, where each example consists of a piece of text and its corresponding sentiment label. The model can then be used to predict the sentiment of new text inputs. One advantage of using GPT-3 for sentiment analysis is that it can generate more natural and coherent language than other traditional supervised learning models, which rely on predefined word embeddings and language rules. Additionally, GPT-3 can capture the context and nuance of the text, allowing it to make more accurate sentiment predictions (Nath et al., 2022). The GPT-3 architecture is based on the Transformer model, which was first introduced by Vaswani et al. (2017). The Transformer model uses a self-attention mechanism that allows it to process input sequences in parallel, rather than sequentially (Kalyan, 2023). This makes it well-suited for processing long sequences of text, which is important for many NLP tasks, including language generation and sentiment analysis as shown in Figure 4.

Unlike other traditional supervised learning models for sentiment analysis, GPT-3 uses a language generation approach, where the model predicts the most likely sequence of words given a prompt. The GPT-3 architecture consists of 175 billion parameters, which is significantly larger than previous versions of the GPT model. The input to the model is a sequence of tokens, which are mapped to high-dimensional embeddings using an embedding layer. The embeddings are then processed by a stack of Transformer encoder layers (Pawar, 2022). Each encoder layer consists of two sub-layers: a multi-head self-attention layer and a position-wise fully connected feed-forward layer. The self-attention layer allows the model to attend to different parts of the input sequence, while the feed-forward layer applies a non-linear transformation to the output of the self-attention layer. In addition to the encoder layers, GPT-3 also includes a task-specific output layer that is used for fine-tuning the model on specific NLP tasks, including sentiment analysis (Nassiri and Akhloufi, 2023).

During fine-tuning, the model learns to predict the sentiment label for each input sequence, given its internal representation. To extract context from the input text, the GPT model uses a combination of weight embeddings and positional embeddings. These embeddings are fed into the multi-head attention layer, which is present in each of the 12 transformer decoder blocks, followed by a feed-forward layer. The softmax function is then applied to the output, generating a probability distribution. Mathematically, this can be expressed in Equations (7–9) as follows (Acheampong et al., 2021):


(7) $$\begin{array}{lll} h_{0} & = & UW_{e}+W_{p} \end{array}$$

(8) $$\begin{array}{lll} h_{l} & = & transformer\_block\left(h_{l-1}\right)\forall i\in \left[1,n\right] \end{array}$$

(9) $$\begin{array}{lll} P\left(u\right) & = & \mathrm{softmax}\left(h_{n}W_{e}^{T}\right) \end{array}$$

The context vector of tokens, represented by U = (u – k, …, u – 1), is used in the GPT model to capture contextual information from neighboring tokens. Here, k represents the context window size, while *W*_*e*_ and *W*_*p*_ represent the token embedding matrix and position embedding matrix, respectively. The GPT model can be pre-trained and fine-tuned for various tasks. By increasing its size, the GPT architecture has been scaled up to create the GPT-2 and GPT-3 models (Pawar, 2022). One advantage of the GPT-3 architecture is its ability to generate coherent and natural language text, making it well-suited for language generation tasks. Additionally, the large number of parameters and layers allows it to capture complex relationships between words and phrases, making it a powerful tool for many NLP tasks. However, the large size and complexity of the model also make it computationally expensive and difficult to fine-tune on smaller datasets. Additionally, the sheer number of parameters means that the model can potentially generate biased or inappropriate language, depending on the training data and prompts used. Moreover, GPT-3 is a proprietary model developed by OpenAI and is not currently available for download (Kalyan, 2023). Therefore, it should be used with caution and with careful prompt optimization to ensure accurate and unbiased results.

### 3.6 Performance metrics

The study utilized several evaluation metrics to assess the performance of the different sentiment analysis models being compared. Accuracy measures the overall proportion of correct sentiment classifications made by the model (Moulaei et al., 2022). It provides a general gauge of the model's capabilities and is calculated in Equation 10 as:


(10) $$\begin{array}{l} \text{Accuracy}=\left(\text{TP}+\text{TN}\right)/\left(\text{TP}+\text{FP}+\text{FN}+\text{TN}\right) \end{array}$$

Where TP = True Positives, TN = True Negatives, FP = False Positives, FN = False Negatives.

Precision measures the proportion of correctly identified positive samples among all samples predicted as positive. This reflects the model's ability to avoid false positives and return mainly relevant results for the positive class. It is defined in Equation 11 as:


(11) $$\begin{array}{l} \text{Precision}=\text{TP}/\left(\text{TP}+\text{FP}\right) \end{array}$$

Recall, also known as sensitivity, measures the proportion of actual positive samples that the model correctly identifies as positive. This reflects the model's ability to capture most of the relevant instances within the positive class. It is calculated according to Equation 12 as follows:


(12) $$\begin{array}{l} \text{Recall}=\text{TP}/\left(\text{TP}+\text{FN}\right) \end{array}$$

Specificity measures the proportion of actual negative samples correctly classified as negative. It evaluates the model's accuracy in predicting the negative class. High specificity indicates the model correctly rejects most irrelevant instances. It is calculated according to Equation 13:


(13) $$\begin{array}{l} \text{Specificity}=\text{TN}/\left(\text{TN}+\text{FP}\right) \end{array}$$

The F1-score is the harmonic mean of precision and recall, providing a balanced measure of the model's precision and recall. It offers a singular metric encapsulating both qualities, and is defined in Equation 14 as:


(14) $$\begin{array}{l} \text{F1-score}=2*\left(\text{Precision}*\text{Recall}\right)/\left(\text{Precision}+\text{Recall}\right) \end{array}$$

These metrics enable thorough quantification of key aspects like precision, recall, and specificity for the sentiment models. The metrics assess the model's exactness, completeness, and effectiveness in identifying the sentiments correctly (Chen et al., 2021). Assessing these characteristics provides comprehensive insights into each model's capabilities and limitations in sentiment analysis, guiding the selection of most suitable models for analyzing tweets. Furthermore, the metrics facilitate standardized comparisons to previous benchmark studies.

### 3.7 Experimental setup

The Bi-LSTM model utilized word-level tokenization along with a text cleaning process that involved replacing emojis, removing special characters and punctuations, hashtags, URLs, stop words, and applying lemmatization and lowercase conversion. Additionally, 200-dimensional GloVe embeddings were used for word representation. The model consisted of 64 LSTM units, a dropout rate of 0.2, and was trained for 50 epochs using binary cross-entropy loss and the Adam optimizer with a learning rate of 0.00001 and a batch size of 32. A tanh activation function was used in the output layer.

In contrast, the BERT model utilized a simpler level of text cleaning by applying lowercasing and removal of special characters, URLs, and hashtags before word-level tokenization. The training process used a learning rate of 0.0005, a batch size of 16, 30 epochs, and the Adam optimizer with a dropout rate of 0.15. The hyperparameter settings for both Bi-LSTM and BERT are shown in Table 1.

**Table 1:** Hyperparameter settings.

**Parameters**	**Bi-LSTM**	**BERT**
Batch size	32	16
Epochs	50	30
Dropout	0.2	0.15
Activation function	Sigmoid	ReLU
Learning rate	0.00001	0.0005

Similarly, GPT-3 employed a simple text cleaning process, removing special characters, URLs, and hashtags from the datasets. The GPT-3 model inherently handles tokenization and generating embeddings without needing explicit stages for these tasks. Prompt engineering was leveraged to format the input text and define the sentiment analysis classification task for GPT-3. Specifically, the text-davinci-003 model was utilized via the OpenAI API with a Temperature of 0.5 to focus on the most probable sentiment classification. While max_tokens parameter was set to a higher value of 50 to allow GPT-3 to generate a complete and meaningful response, the output was post-processed to extract only the first word, ensuring that the sentiment is categorized as positive, negative, or neutral. Moreover, the parameter n was set to 1 specifying the number of independent completions to generate from the same prompt. The designed prompt was chosen to be “Please analyze the sentiment (with only positive or negative) of the following text: {text}” for binary sentiments datasets as in the utilized standard datasets. While for three sentiments datasets we used the prompt “Please analyze the sentiment (with only positive or negative or neutral) of the following text: {text}.” To adhere to OpenAI's API key usage limits, a one-second delay was introduced between processing each tweet and obtaining a response.

## 4 Results and discussions

### 4.1 Data visualization

**Figure 5:**
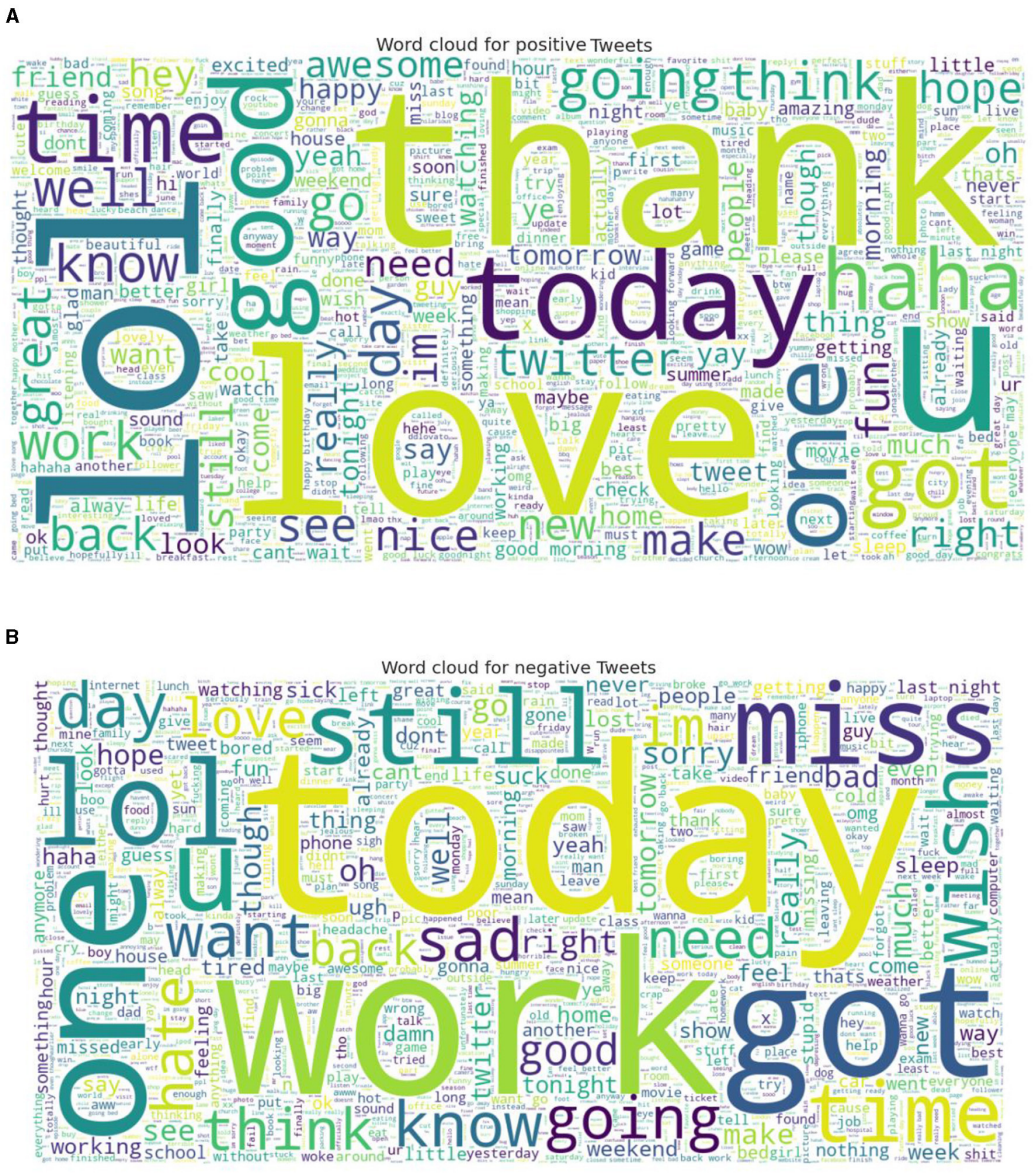
Word cloud for Sentiment140 for both **(A)** Positive, and **(B)** Negative sentiments.

**Figure 6:**
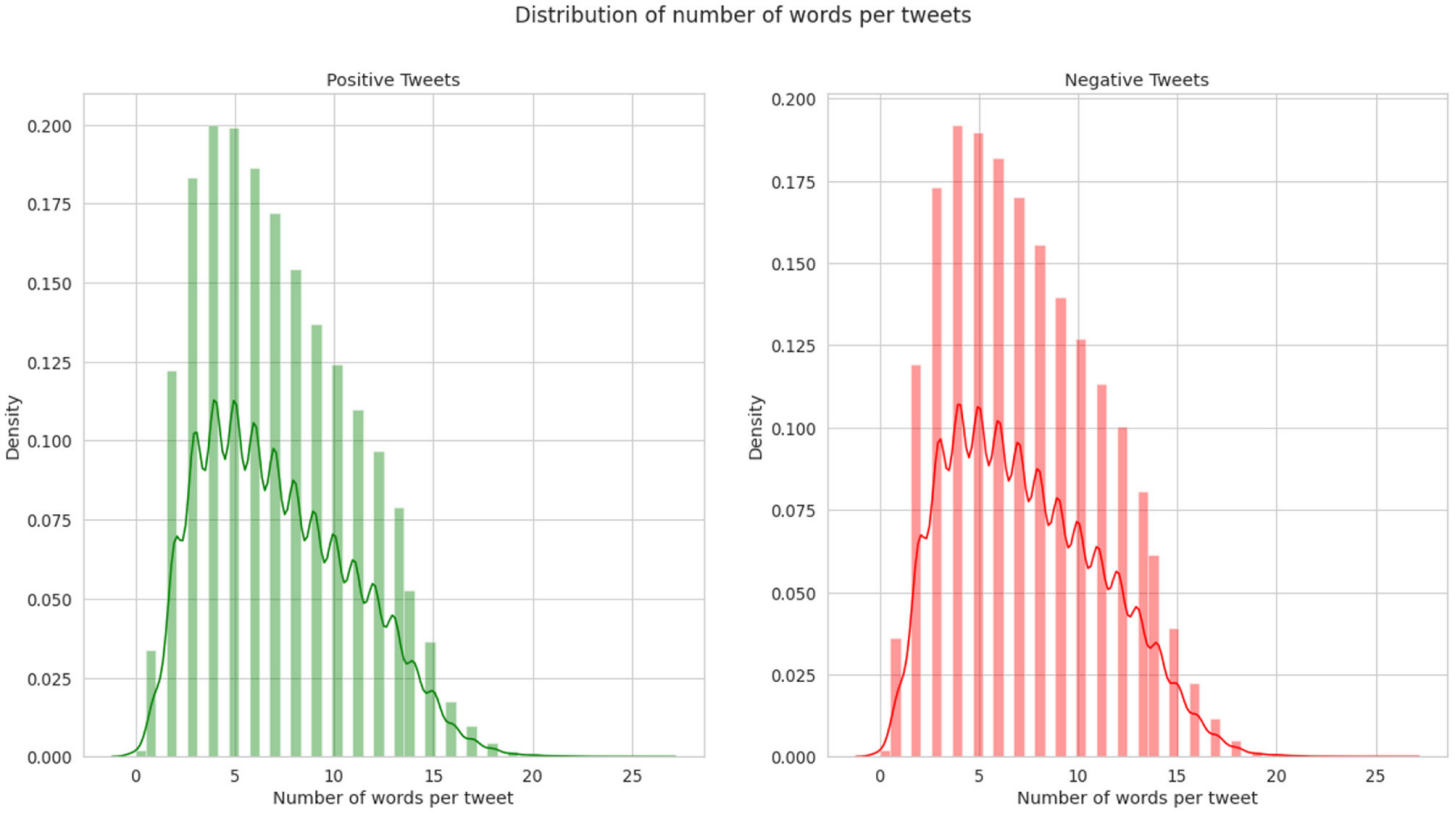
Average number of words per tweet of Sentiment140.

**Figure 7:**
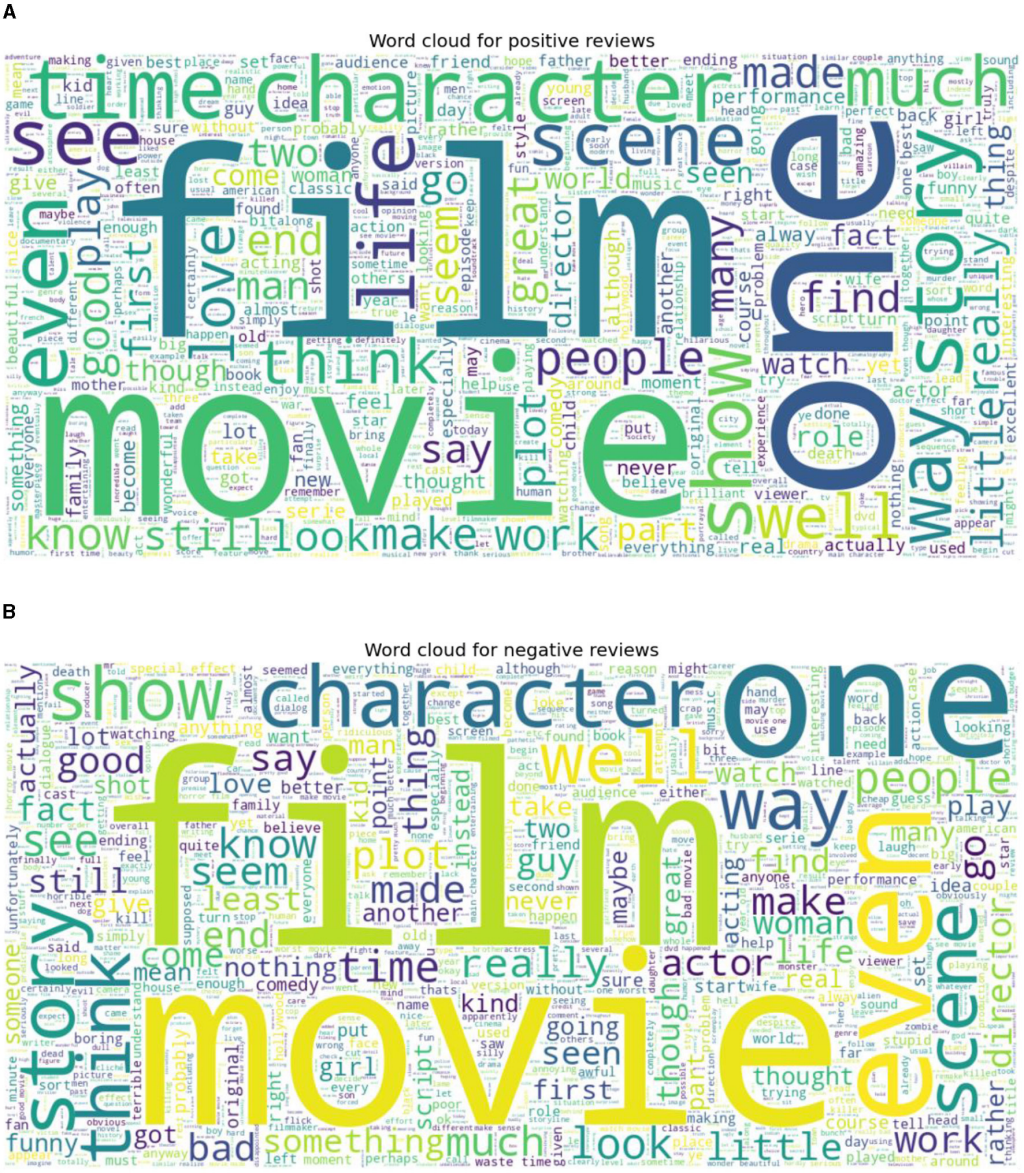
Word cloud for IMDB review for both **(A)** Positive, and **(B)** Negative sentiments.

**Figure 8:**
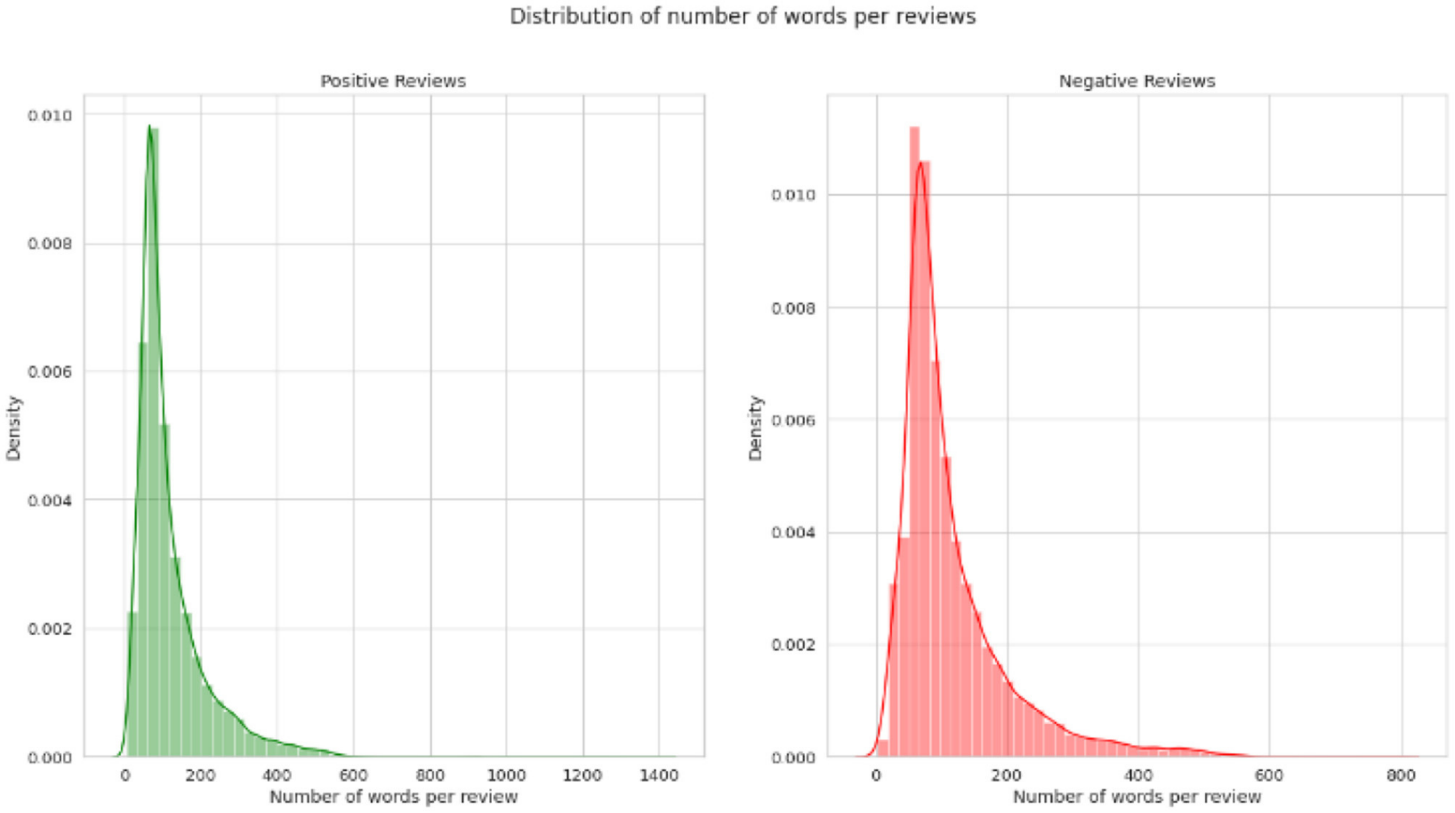
Average number of words per tweet of IMDB review.

Two standard datasets were used to evaluate the sentiment analysis techniques proposed in this research. The first one is Sentiment140 with binary sentiments of positive and negative as shown in Figure 5. It has much shorter tweets and higher number of data samples than IMDB reviews as shown in Figure 6. While the IMDB reviews dataset similarly has binary sentiments of positive and negative as shown in Figure 7 with relatively longer text and fewer number of data samples as shown in Figure 8. Shorter text will mean more difficult for the sentiment analysis model to achieve high accuracy in detecting the right sentiment. Therefore, both were necessary to compare the performance of the different approaches used in NLP.

**Figure 9:**
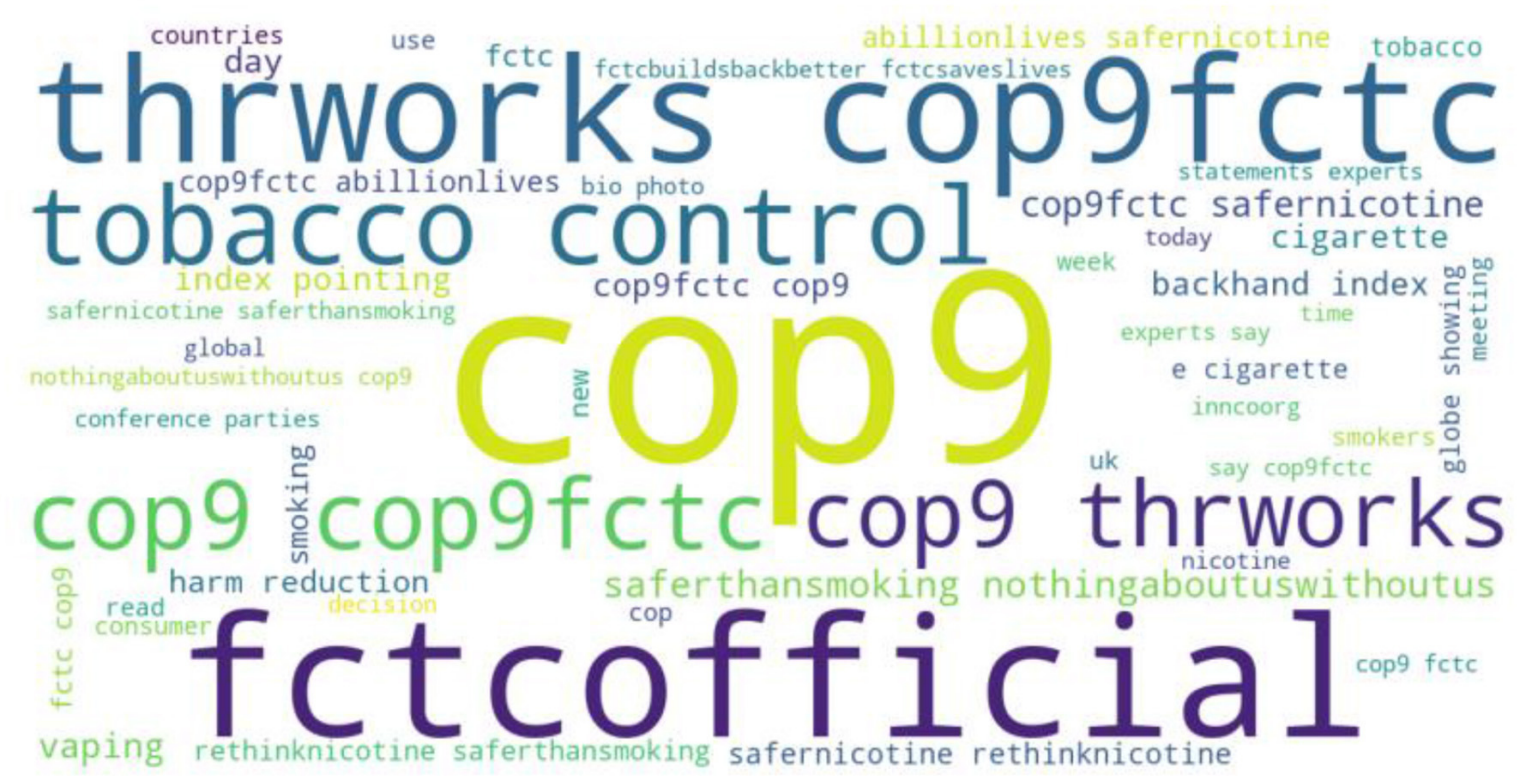
Word cloud for COP9 tweets.

On the other hand, the third dataset is COP9 tweets with three sentiments namely, negative, neutral, and positive. The dataset word cloud is shown in Figure 9.

### 4.2 First stage

The study started with Vader Lexicon sentiment analyser that was applied to all the reviews and tweets in IMDB and Sentiment140. A text cleaning of lowercasing, Emoji replacement, removing URL and Hashtags from the text was performed before applying the Lexicon rules. However, Vader achieved moderate performance of F1-Score 0.64 and 0.56 for both IMDB and Sentiment140 respectively. Because Vader does not rely on training, it is not specific to the theme of the datasets.

A variety of machine learning techniques, including Logistic Regression, Naïve Bayes, SVM, XGBoost, Extra Trees, Random Forest, and K-Nearest Neighbors (KNN), were employed with an 80% training and 20% testing split for both datasets. Furthermore, a 10-fold cross-validation procedure was conducted to assess the model performance. Preceding the application of these Machine Learning techniques, the datasets underwent thorough text cleaning and word-level tokenization processes. These steps encompassed emoji replacement, elimination of special characters and punctuation, removal of hashtags and URLs, exclusion of stop words, lemmatization, and conversion to lowercase. Subsequently, features were extracted using TF-IDF.

These sentiment models were trained and tested using IMDB reviews dataset with an F1-Score performance of a maximum value of 0.89 achieved using logistic regression and TF-IDF as shown in Table 2. The same sentiment models were trained and tested using Sentiment140. The results achieved a lower F1-Score performance with a maximum value of 0.76 as shown in Table 3.

**Table 2:** The performance of the sentiment analysis models based on IMDB review dataset.

**Model**	**Accuracy**	**Precision**	**Sensitivity**	**Specificity**	**F1-Score**
VADER	0.6963	0.5363	0.7886	0.6487	0.6384
Logistic regression	0.8903	0.8737	0.9029	0.8786	0.8880
Naive bayes	0.8679	0.8735	0.8630	0.8729	0.8682
SVM	0.8957	0.8816	0.9065	0.8856	0.8939
XGboost	0.8463	0.8188	0.8654	0.8293	0.8414
Extra trees	0.8660	0.8579	0.8711	0.8611	0.8644
Random forest	0.8612	0.8534	0.8660	0.8566	0.8597
KNN	0.7733	0.6999	0.8186	0.7397	0.7546
Bi-LSTM	0.8827	0.9281	0.8496	0.9220	0.8971
BERT	0.9380	0.9529	0.9236	0.9529	0.9380
GPT-3	0.9076	0.9260	0.8750	0.9469	0.9119

**Table 3:** The performance of the sentiment analysis models based on Sentiment140 dataset.

**Model**	**Accuracy**	**Precision**	**Sensitivity**	**Specificity**	**F1-Score**
VADER	0.6615	0.4248	0.8066	0.6096	0.5565
Logistic regression	0.7675	0.7495	0.7771	0.7586	0.7631
Naive bayes	0.7472	0.7950	0.7252	0.7737	0.7585
SVM	0.7654	0.7519	0.7724	0.7588	0.7620
XGboost	0.7326	0.6484	0.7792	0.6995	0.7078
Extra trees	0.7602	0.7719	0.7540	0.7668	0.7628
Random forest	0.7458	0.7712	0.7321	0.7608	0.7511
KNN	0.5511	0.9490	0.5271	0.7567	0.6778
Bi-LSTM	0.7706	0.7550	0.7809	0.7608	0.7778
BERT	0.8254	0.7513	0.8818	0.7835	0.8114
GPT-3	0.7911	0.7913	0.7913	0.7908	0.7913

The Bi-LSTM and BERT models were evaluated on the IMDB and Sentiment140 datasets using a 70:10:20 split for training, validation, and testing, respectively. The Bi-LSTM model resulted in slightly improved performance, achieving F1-Score values of 0.90 and 0.78 for the IMDB and Sentiment140 datasets, respectively. While BERT achieved the highest F1-Score values of 0.94 and 0.81, corresponding to the IMDB and Sentiment140 datasets, respectively. Furthermore, owing to its pretraining, GPT-3 required minimal configuration and did not rely on any specific datasets. Nevertheless, it achieved relatively good F1-Score performance of 0.91 and 0.79 when applied to IMDB and Sentiment140, respectively. Thus, the results achieved relatively better F1-Score than all other sentiment models except BERT for the utilized standard datasets.

The superior performance of BERT and GPT-3 compared to machine learning models can be attributed to their large pre-trained language models, which provide richer representations of text. Their bidirectional context and transformer architectures allow modeling intricate dependencies between words and sentences. BERT's fine-tuning directly on the sentiment analysis datasets enables it to learn nuanced expressions of sentiment from the data. In contrast, machine learning models rely on feature engineering, which may not fully capture semantic complexities. The informal language and shorter length of tweets likely impacted machine learning models more than BERT and GPT-3. The latter's pre-training makes them more adaptable to diverse text types. GPT-3′s generative capabilities could allow it to better compose coherent sentiments despite limited data, explaining its resilient performance even with no dataset training. Factors like model architecture, handling of informal text, capability to learn contextual relationships, and transfer learning abilities contributed to the variance in performance across techniques.

### 4.3 Second stage

**Figure 10:**
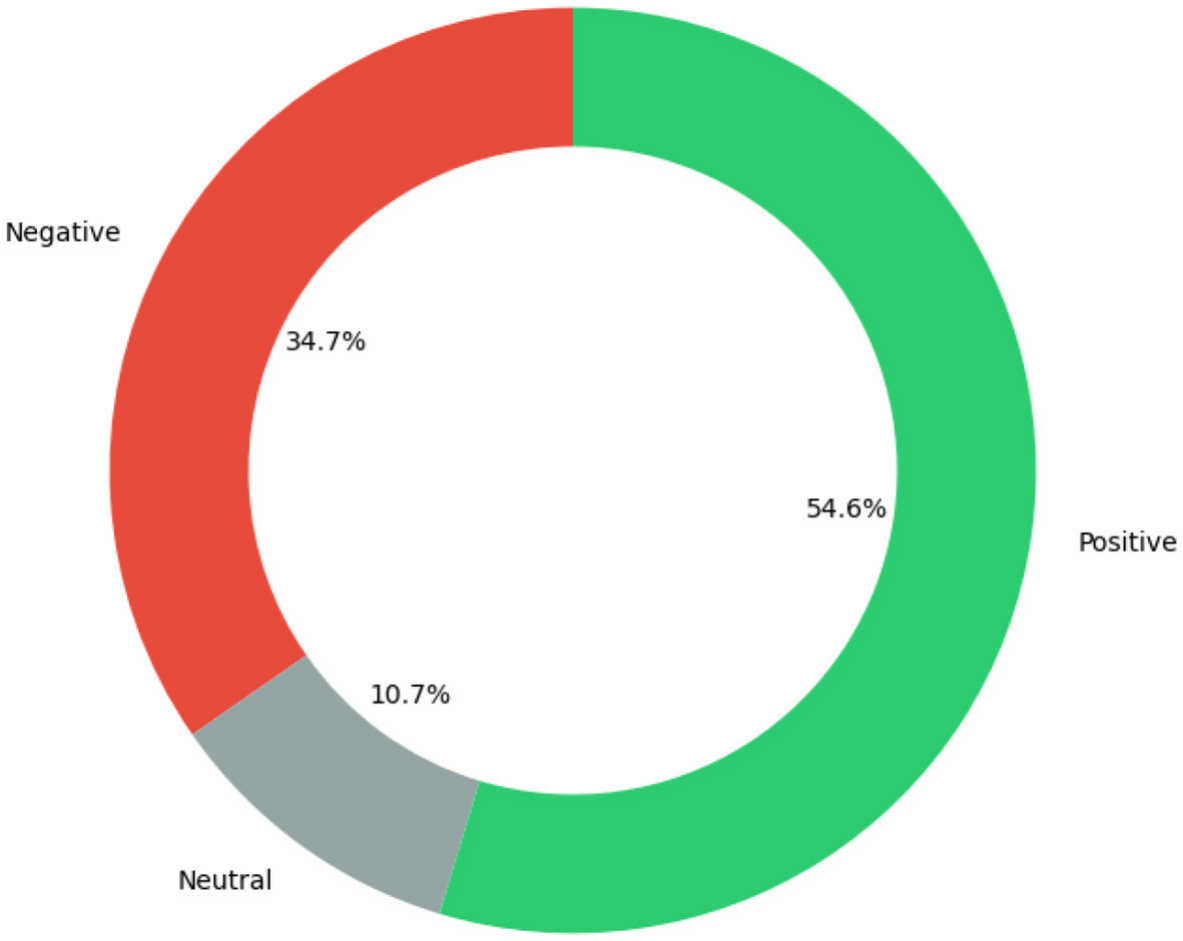
Sentiment distribution for the whole COP9 tweets.

The second stage of analysis used Vader, Bi-LSTM, BERT, and GPT-3 as they are suitable for three sentiment analysis with little or no adjustment. In addition, Bi-LSTM, BERT, and GPT-3 have achieved best performance with both IMDB and Sentiment140. Therefore, they were applied to the partially annotated tweets from COP9 tweets. All the applied sentiment models were trained based on IMDB reviews and Sentiment140. The applied sentiment analysis models' performance was summarized in Table 4. Among the evaluated models, GPT-3 achieved the highest overall accuracy on the partially annotated COP9 dataset. To gain deeper insights, we further analyzed its performance metrics for each sentiment category (Negative, Neutral, Positive) using the same partially annotated COP0 dataset. As shown in Table 5, GPT-3 exhibited good performance across all classes, with precision values ranging from 81% to 87% and F1-scores between 78% and 89%. Therefore, GPT-3 was applied to the whole COP9 tweets which resulted in sentiments distribution with higher positive tweets than both negative and neutral sentiments as shown in Figure 10. This sentiment distribution provides a promising indication regarding the sentiment surrounding the COP9 conference.

**Table 4:** Sentiment analysis models performance with the partially annotated COP9 tweets.

**Model**	**Accuracy^a^**	**Accuracy^b^**
VADER	0.5831	0.5831
Bi-LSTM	0.8412	0.7523
BERT	0.8627	0.8141
GPT-3	0.8812	0.8812

^a^Denotes sentiment models trained using IMDB review while ^b^are the sentiment models trained using Sentiment140.

**Table 5:** Performance metrics (precision, recall, and F1-score) of the GPT-3 sentiment analysis for the partially annotated COP9 tweets.

	**Precision**	**Recall**	**F1-score**
Negative	85%	74%	79%
Neutral	81%	75%	78%
Positive	87%	91%	89%

## 5 Conclusions

Based on the achieved results, BERT and GPT-3 are highly effective in sentiment analysis when used with the IMDB reviews dataset. This could be attributed to the fact that the dataset has long tweets which can clearly express sentiments. Moreover, BERT and GPT-3 do not require extensive data cleaning as compared to Bi-LSTM and ML, which can lead to better performance. While for short texts such as those found in Sentiment140 and tweets, BERT performed better than other techniques as it was specifically tuned on the Sentiment140 dataset.

One of the challenges in sentiment analysis is dealing with imbalanced data, where the number of samples in one class is significantly larger than the other. However, GPT-3 is not susceptible to such problems as other sentiment analysis models and can deal with multilingual text. Besides, GPT-3 could achieve F1-Score performance ranging from 0.8 for short text to 0.9 for longer text. That performance is consistent and comparable to most of the techniques that require prior training using the dataset under consideration. In addition, given that GPT-3 has already been pre-trained, it does not need a complex training procedure to be applied to the specific dataset. This makes it ideal for real-time sentiment analysis or in cases where it is difficult to annotate data. Future research could explore the potential of leveraging other foundation models for sentiment analysis. As the field of NLP keeps advancing, several powerful pre-trained models like LLAMA, Flan-T5, and Falcon (Penedo et al., 2023; Gu et al., 2024; Roumeliotis et al., 2024) have emerged, providing diverse capabilities for comprehending and analyzing textual data. Additionally, fine-tuning techniques such as LoRA (Ge et al., 2023) substantially reduce the number of trainable parameters by incorporating a small number of new weights into the model and exclusively training those parameters, demonstrating promising results.

Building on the promising performance of models like GPT-3 for general sentiment analysis, its potential in analyzing sentiments for domain-specific content related to the COP9 tobacco control conference was further explored. The prevalence of positive polarity was observed, indicating that the conference content and discussion topics were well-aligned with the opinions and preferences of a majority of attendees based on their expressed sentiment. However, further analysis would be needed to correlate specific conference aspects to the observed sentiment trends to objectively identify strengths, weaknesses, and areas for improvement. Quantitatively tracking sentiment over conferences provides data that organizers could potentially leverage to assess engagement, satisfaction, and other factors. Additionally, analyzing trends in tobacco-related tweet sentiment enables identification of prevailing public opinions, which could ultimately inform education campaigns or advocacy efforts. However, definitive claims about the degree of influence on participation, policy or advocacy require further controlled studies. Therefore, sentiment analysis of domain-specific social media data provides valuable feedback and insights, but conclusions should be drawn with objectivity and caution against overgeneralization.

## Data availability statement

The data presented in the study are deposited in the Harvard Dataverse repository, accession number https://doi.org/10.7910/DVN/UILQHY.

## Author contributions

SE: Writing – review & editing, Writing – original draft, Visualization, Validation, Software, Methodology, Data curation, Conceptualization. JM: Writing – review & editing, Writing – original draft, Visualization, Validation, Supervision, Project administration, Methodology, Investigation, Formal analysis.
